# A method for mining condition-specific co-expressed genes in *Camellia sinensis* based on k-means clustering

**DOI:** 10.1186/s12870-024-05086-5

**Published:** 2024-05-08

**Authors:** Xinghai Zheng, Peng Ken Lim, Marek Mutwil, Yuefei Wang

**Affiliations:** 1 Tea Research Institute, Zhejiang University, Hangzhou, 310058 Zhejiang China; 2 School of Biological Sciences, Nanyang Technological University, 60 Nanyang Drive, Singapore, 637551 Singapore

**Keywords:** Condition-specific gene interactions, Gene co-expression network analysis, K-means clustering, Correlation difference value, Sustained cold stress

## Abstract

**Background:**

As one of the world’s most important beverage crops, tea plants (*Camellia sinensis*) are renowned for their unique flavors and numerous beneficial secondary metabolites, attracting researchers to investigate the formation of tea quality. With the increasing availability of transcriptome data on tea plants in public databases, conducting large-scale co-expression analyses has become feasible to meet the demand for functional characterization of tea plant genes. However, as the multidimensional noise increases, larger-scale co-expression analyses are not always effective. Analyzing a subset of samples generated by effectively downsampling and reorganizing the global sample set often leads to more accurate results in co-expression analysis. Meanwhile, global-based co-expression analyses are more likely to overlook condition-specific gene interactions, which may be more important and worthy of exploration and research.

**Results:**

Here, we employed the k-means clustering method to organize and classify the global samples of tea plants, resulting in clustered samples. Metadata annotations were then performed on these clustered samples to determine the “conditions” represented by each cluster. Subsequently, we conducted gene co-expression network analysis (WGCNA) separately on the global samples and the clustered samples, resulting in global modules and cluster-specific modules. Comparative analyses of global modules and cluster-specific modules have demonstrated that cluster-specific modules exhibit higher accuracy in co-expression analysis. To measure the degree of condition specificity of genes within condition-specific clusters, we introduced the correlation difference value (CDV). By incorporating the CDV into co-expression analyses, we can assess the condition specificity of genes. This approach proved instrumental in identifying a series of high CDV transcription factor encoding genes upregulated during sustained cold treatment in *Camellia sinensis* leaves and buds, and pinpointing a pair of genes that participate in the antioxidant defense system of tea plants under sustained cold stress.

**Conclusions:**

To summarize, downsampling and reorganizing the sample set improved the accuracy of co-expression analysis. Cluster-specific modules were more accurate in capturing condition-specific gene interactions. The introduction of CDV allowed for the assessment of condition specificity in gene co-expression analyses. Using this approach, we identified a series of high CDV transcription factor encoding genes related to sustained cold stress in *Camellia sinensis*. This study highlights the importance of considering condition specificity in co-expression analysis and provides insights into the regulation of the cold stress in *Camellia sinensis*.

**Supplementary Information:**

The online version contains supplementary material available at 10.1186/s12870-024-05086-5.

## Introduction

As one of the most popular non-alcoholic beverages worldwide, tea contains a wide range of secondary metabolites beneficial to human health, such as polyphenols, alkaloids, and theanine [1]. As such, the tea plant (*Camellia sinensis*) possesses a diverse range of germplasm resources [2]. Different cultivars of *Camellia sinensis *are each prized for certain desirable qualitiesin their own right and exhibit significant differences in plant morphology, leaf characteristics, growth habits, adaptability, and secondary metabolites [3, 4]. Consequently, said tea cultivars have garnered much research interest in the post-genomic era to understand and improve tea traits.

With an increasing number of studies on the epigenetic variations and compositional changes of secondary metabolites in tea plants under different experimental conditions [1, 4, 5], the omics dataset of *Camellia sinensis *has also become increasingly extensive. This has led to the use of systems biology approaches on sequencing data hosted on public databases [6–8], such as gene co-expression analysis, becoming a trend in analyzing omics data of *Camellia sinensis*, providing tea researchers with a more macroscopic and comprehensive perspective. Researchers have further downloaded large-scale transcriptome data of tea plants and created a more systematic and comprehensive co-expression database TeaCoN (http://teacon.wchoda.com) [9].

Although the large sample size of publicly-derived *Camellia sinensis* transcriptomic data improves the statistical significance of relationships between genes and increases the reliability of inferring gene correlations, indiscriminately combining multiple samples may not be universally beneficial [10]. As datasets become larger and more diverse, the derived coexpression networks become less informative due to increased multidimensional noise [11]. One way to improve the utility of the network is downsampling. Downsampling subdivides samples either by manually grouping them based on experimental conditions or by using automated methods such as k-means clustering [12–14]. However, manual grouping often lacks sufficient sample description to accurately classify them, so automated methods like k-means clustering are more effective [12].

Furthermore, co-expression networks at a large scale of samples may miss specific gene interactions formed under particular conditions [15]. Increasing evidence suggests that different gene networks operate in different biological contexts [16, 17]. Therefore, it becomes increasingly important to compare and contrast coexpression networks under specific conditions [18–20]. Experimental results demonstrate that over one-third of genetic interactions are condition-specific [21]. Several studies have also shown that the patterns of gene coexpression vary under different conditions [22–24]. Hence, when conducting coexpression analysis on large-scale samples, incorporating sample auto-classification and mining condition-specific coexpressed genes can enhance the accuracy and informativeness of co-expression analysis.

In this study, all *Camellia sinensis* samples downloaded from NCBI were subjected to k-means clustering to obtain four clusters representing different “conditions” (experimental treatments, tissues, and cultivars). Cluster metadata annotations were obtained through sample metadata annotation. Then, weighted gene co-expression network analysis (WGCNA) was performed on the expression profiles of both the global samples and the cluster samples to obtain their respective co-expression modules. Subsequently, the correlation difference value (CDV) was proposed to measure the degree of condition specificity of genes within condition-specific clusters. By comparing between clusters and within clusters, highly condition-specific clusters and biological functions were identified. By incorporating the CDV into gene regulatory networks and visualizing it, condition-specific genes and conserved genes can be distinguished, providing more information for the selection of key genes. Overall, this study aims to improve gene co-expression analysis methods for large-scale transcriptomic data of tea plants by performing condition-specific analysis and providing a more accurate understanding of the relationships between gene expression patterns and phenotypic traits.

## Methods

### Data sources and sample metadata annotation

By searching and filtering using the keyword “Camellia sinensis” in the NCBI SRA database, a total of 760 RNA-Seq raw reads were obtained. The initial annotation of these RNA-Seq raw data were performed, selecting the relatively important metadata fields in the NCBI SRA database for *Camellia sinensis* research, including cultivar, plant tissue, and experimental treatments. Subsequently, the corresponding original papers for each RNA-Seq data were searched to retrieve annotation information (Table S1).

To facilitate differentiation from other experimental treatments, the control group and samples directly collected without any treatment were uniformly labeled as “no treatment” in the experimental treatment column. The samples with missing annotations in the metadata fields of the NCBI SRA database and could not be found in the retrieved original papers were labeled as “missing”.

### Expression quantification and gene functional annotation

Seven hundred sixty RNA-seq samples were processed using fastp tool [25] to obtain high-quality clean data by removing adapter sequences and low-quality reads using default parameters. Coding sequences (CDS) annotations of the “Shuchazao” *Camellia sinesis* cultivar (http://tpia.teaplant.org) [26] were used as pseudoalignment reference, the processed reads were then used to quantify the gene expression, in transcripts per million (TPM) values, for all RNA-seq samples using Kallisto [27] (Table S2).

The CDS annotations of the tea plant cultivar “Shuchazao” were subjected to gene functional annotation using the Mercator v4 6.0 [28] (Table S3).

### K-means clustering and cluster metadata annotation

Firstly, 16,094 genes were selected from the CDS of "Shuchazao" whose average expression levels were greater than 2 in 760 samples and were annotated with detailed biological functions by Mercator v4 6.0. Then, a gene expression profile was constructed using TPM values of 16,094 genes from 760 RNA-seq samples. The batch effects in the gene expression profile were reduced by normalizing the expression levels using the StandardScaler tool from the sklearn.preprocessing package. The KMeans tool from the sklearn.cluster package was used for k-means clustering on all RNA-seq samples with a random seed set to 1024 (np.random.seed(1024)) and a target number of clusters set to 4 (n_clusters=4) [29]. The selection of 4 as the value of k in k-means clustering is based on the Silhouette plot, where 4 resulted in a better classification of the samples (Figure S1) [30].

Then, the TSNE tool from the sklearn.manifold package was applied to the standardized gene expression profile to perform dimensionality reduction, retaining the top principal components Component 1 and Component 2. Finally, the samples were visualized in the Component 1 and Component 2 space to explore potential clustering structures and similarities among the samples, as described by [31].

To annotate the 4 clusters obtained from k-means clustering, the hypergeom tool from the scipy.stats package was used to perform a hypergeometric test between each sample in each cluster and the samples associated with each cultivar term [32]. Then, the fdrcorrection tool from the statsmodels.stats.multitest package was used to correct the p-values of all cultivar terms corresponding to each cluster, obtaining the false discovery rate (FDR) values [33]. Cultivar terms with FDR values less than or equal to 0.05 were selected as metadata annotations for the samples in that cluster. The same approach was applied to obtain metadata annotations for the tissue terms and experimental treatment terms (Figure S2; Table S4).

### Weighted gene co-expression network analysis (WGCNA)

The global expression profile and cluster expression profiles comprise the expression levels of 16,094 genes from global samples and samples from different clusters (cluster samples), respectively. 

R package WGCNA was then employed to construct a co-expression network [34]. WGCNA was performed following the guidelines provided in the tutorial (https://horvath.genetics.ucla.edu/html/CoexpressionNetwork/Rpackages/WGCNA/Tutorials/) [35], where employing a stepwise approach to divide the modules based on the obtained soft threshold and utilized the DynamicTreecut package to cluster the modules.

The eigengenes of each module were calculated based on the expression profiles and module color codes. Eigengenes represent the main expression patterns of each module and can be used to describe the overall expression patterns of the module. Then, hierarchical clustering (average linkage) was applied to cluster the module eigengenes. A merging height threshold of 0.25 was set, corresponding to a correlation threshold of 0.75, and called the mergeCloseModules function for automatic module merging. The merged modules were assigned new color codes, which served as the final module color codes (Figure S2). For easy reference, the color modules were mapped to alphabetical letters (Table S5).

In this step, the co-expression modules obtained from the global expression profile are referred to as “global modules”, while the co-expression modules obtained from the cluster expression profiles are referred to as “cluster-specific modules”

### Calculation of clustering similarity and gene condition specificity

The Fowlkes-Mallows score (FMS) and adjusted mutual information score (AMIS), which is used to analyze the similarity of co-expression modules in WGCNA under different samples, was calculated using the fowlkes_mallows_score and adjusted_mutual_info_score tool from the sklearn.metrics package [36, 37].

Then, for each cluster, the two kinds of gene-module consistency coefficient (GMC) were calculated for each gene - the GMC of the gene in the cluster-specific module and the GMC of the gene in the global module based on corresponding cluster samples. The GMC of a gene is defined as the Pearson correlation coefficient (PCC) between the gene’s expression profile and the module eigengene expression profile of the co-expression module it belongs to [38]. The mathematical formula for GMC is as follows:

$$GMC=\frac{{\displaystyle\sum_i^n}\left(g_i-\overline g\right)\left(eg_i-\overline{eg}\right)}{\sqrt{\displaystyle\sum_i^n}\left(g_i-\overline g\right)^2{\displaystyle\sum_i^n}\left(eg_i-\overline{eg}\right)^2}$$

where *g*_*i*_ and e.g._*i*_ represent the expression values of gene and module eigengene in the *i*-th sample, `*g* and `e.g. represent the average expression values of gene and module eigengene, and *n* is the number of samples. This study used the GMC of genes to investigate the consistency of gene expression patterns within co-expression modules.

Furthermore, for each cluster, the correlation difference value (CDV) of each gene was calculated by subtracting the GMC of the gene in the cluster-specific module from the GMC of the gene in the global module. The mathematical formula for CDV is as follows:

$$CDV=\frac{{\displaystyle\sum_i^m}\left(g_i-\overline g\right)\left(ceg_i-c\overline eg\right)}{\sqrt{\displaystyle\sum_i^m}\left(g_i-\overline g\right)^2{\displaystyle\sum_i^m}\left(ceg_i-c\overline eg\right)^2}-\frac{{\displaystyle\sum_i^m}\left(g_i-\overline g\right)\left(geg_i-g\overline eg\right)}{\sqrt{\displaystyle\sum_i^m}\left(g_i-\overline g\right)^2{\displaystyle\sum_i^m}\left(geg_i-g\overline eg\right)^2}$$

where ce.g._*i*_ and ge.g._*i*_ represent the expression values of eigengene in the cluster-specific module and global module in the *i*-th sample, `*ceg* and `*geg* represent the average expression values of eigengene in the cluster-specific module eigengene and global module, and *m* is the number of samples in the cluster. In this study, CDV was used to measure the condition-specificity of each gene. The genes with only GMC(Cluster) greater than or equal to 0.6 and GMC(Global) greater than or equal to 0.6 were used to calculate the CDV value.

### Functional enrichment analysis of co-expression modules

To annotate the functions of modules, the hypergeometric tests [32] were performed using the hypergeom tool from the scipy.stats package for each gene in the module and each gene included in the mapman entries. To ensure the displayed mapman entries are as detailed as possible, covering all biological functions and with a substantial number of genes, only mapman entries with detailed classification and containing more than 100 genes are selected. Then, the p-values of all mapman entries corresponding to each module are corrected using the fdrcorrection tool from the statsmodels.stats.multitest package to obtain FDR values [33]. Mapman entries with FDR values less than or equal to 0.05 are considered functional annotations for the genes in that module (Table S6).

### Construction of the gene regulatory network

Based on module correlation and module functional annotation, the module associated with the metadata annotation of the cluster was selected. R package GENIE3 was then employed to predict the regulatory relationships between genes within the module [39]. To construct the gene regulatory network, only gene pairs with a weight value greater than or equal to 0.06 were considered.

The constructed gene regulatory networks were imported into Cytoscape software [40] for visualization and analysis. Finally, the network was further customized with layout, labeling, and color coding to provide a clearer representation of the interactions between genes to understand the structure and function of the gene network and to reveal important associations and regulatory mechanisms in biological processes.

## Results

### Metadata annotation of RNA-Seq samples exhibits an imbalanced distribution characteristic

**Fig. 1:**
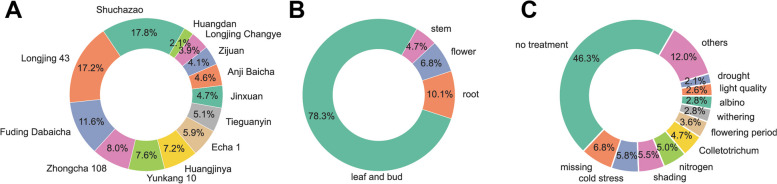
Analysis of metadata for RNA-seq samples of *Camellia sinensis*. **A** Cultivar. **B** Tissue. **C** Experimental treatments

During the metadata annotation of 760 RNA-seq samples of *Camellia sinensis*, we observed that there was an imbalance in the sample distribution across each metadata category, including cultivars, tissues, and treatments (Fig. 1; Table S1). Specifically, certain categories within tissues and treatments have a higher number of samples compared to others. For example, in the 760 *Camellia sinensis* RNA-seq samples, the “leaf and bud” samples accounted for 78.3% of the total, while the “no treatment” samples in the experimental treatments category accounted for 46.3%, far exceeding the numbers of other categories (Fig. 1). Regarding cultivars, we saw a relatively balanced representation across different categories, but some cultivars have a higher proportion. For instance, “Shuchazao” accounts for 17.8%, “Longjing 43” accounts for 17.2%, and “Fuding Dabaicha” accounts for 11.6% (Fig. 1).

### K-means clustering effectively classified global samples and significantly improved the accuracy of co-expression analysis

K-means clustering is used to organize and classify the globally imbalanced samples in the metadata term. The metadata annotations of the clustered samples are then used as the “conditions” representing the specificity of the cluster samples.

In this study, the silhouette score was used to determine the value of K in k-means clustering. The method of selecting the appropriate K value using the silhouette score primarily considers two indicators: (1) For a particular K value, all clusters should have a silhouette score higher than the average score of the dataset, as represented by the red-dotted line on the x-axis. Clusters with K values of 3, 5, 6, 10, and 11 are eliminated because they do not meet this condition (Figure S1). (2) There should not be significant fluctuations in the cluster sizes. The width of the clusters corresponds to the number of sample points. Only K values of 2 and 4 exhibit relatively uniform widths (Figure S1). Here, we chose 4 as the K value.

**Fig. 2:**
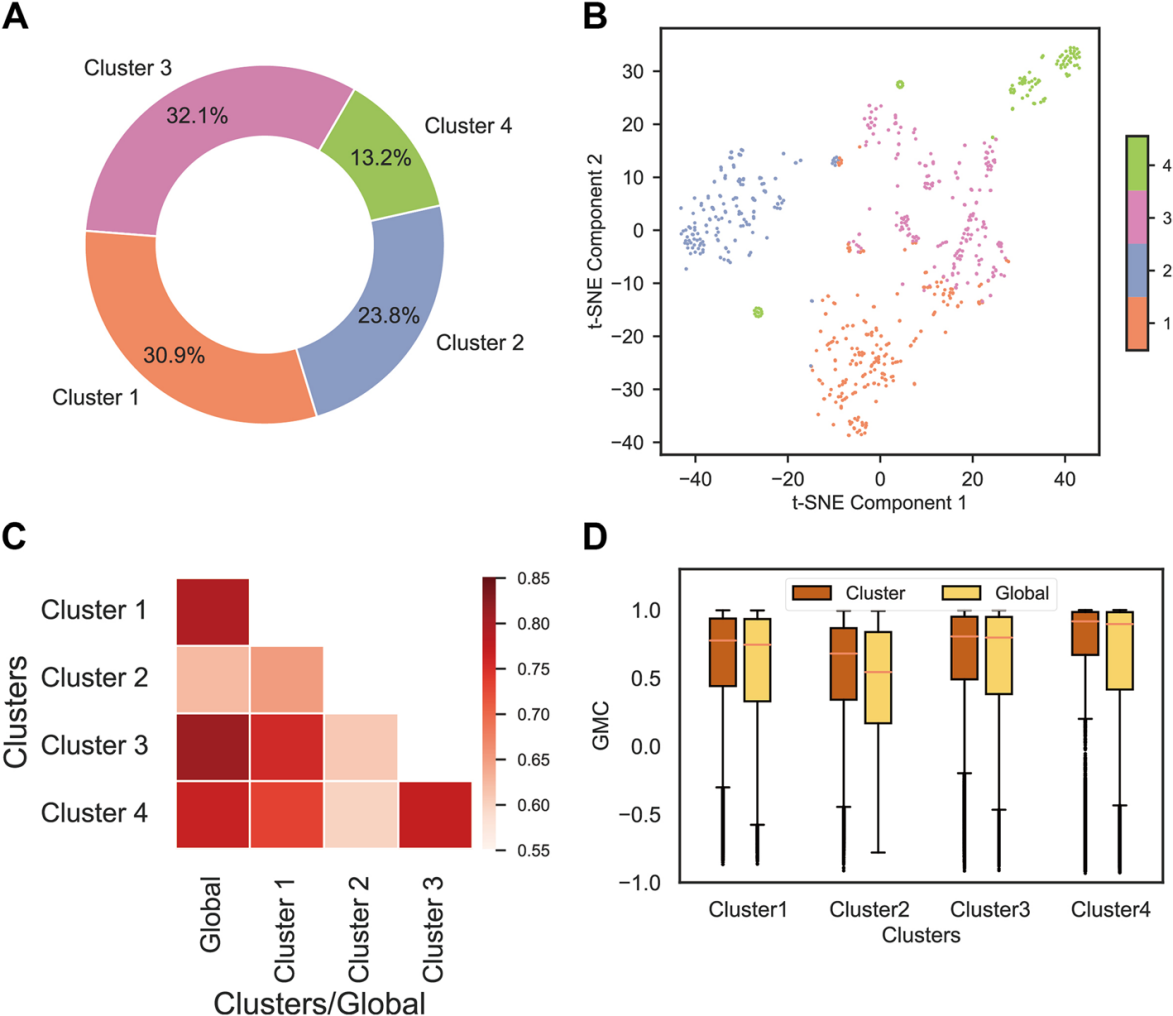
K-means clustering of *Camellia sinensis* RNA-seq samples and comparative analysis of global vs. cluster-specific co-expression modules. (A) Pie chart showing the proportion of k-means clusters. (B) Scatter plots of t-SNE show the spatial distribution of all *Camellia sinensis* RNA-seq samples on Component 1 and Component 2. Different clusters are distinguished using different colors, while the same cluster remains consistent across A and B. (C) Similarity analysis of global and cluster-specific co-expression modules. The intensity of colors in the heatmap represents the magnitude of the Fowlkes-Mallows score (FMS). (D) Comparison of the gene-module consistency coefficient (GMC) of all genes between the global module and the cluster-specific module for each cluster

After analyzing 760 *Camellia sinensis* RNA-seq samples using the k-means clustering algorithm, four clusters were obtained, with Cluster 1 to Cluster 4 accounting for 30.9% (235 samples), 23.8% (181 samples), 32.1% (244 samples), and 13.2% (100 samples) of the total samples, respectively (Fig. 2A) (Table 1). By performing t-Distributed Stochastic Neighbor Embedding (t-SNE) on the transcriptome data to reduce the dimensionality of the genes, the spatial distribution of these samples in Component 1 and Component 2 was observed, where samples from the 4 clusters were separated (Fig. 2B).

**Table 1:** Metadata annotation table of k-means clusters

Clusters	Cultivar	Tissue	Treatment	# Modules	# Samples
Cluster 1	Huangjinya	leaf and bud	shading, MeJA, Colletotrichum, withering, fluoride, salicylic acid, sucrose	12	235
Cluster 2	Longjing 43, Shuchazao	leaf and bud	cold stress, shading	23	181
Cluster 3	Echa 1, Jinxuan, Longjing 43	flower, stem	cold stress, NAA, selenite, insect infestation, mechanical damaged, UV-B, flowering period	22	244
Cluster 4	Anji Baicha, Fuding Dabaicha, Longjing Changye, Zhongcha 108	root	albino, selenite, As, Cd, Na_2_SeO_3_	14	100

By conducting enrichment analysis on the metadata of k-means clusters, metadata terms related to cultivars, tissues, and treatments were annotated to each k-means cluster, facilitating a better understanding of the characteristics and functions of *Camellia sinensis* RNA-seq samples represented by each k-means cluster (Table 1). For example, Cluster 2 mainly includes leaves and buds of the “Longjing 43” and “Shuchazao” cultivar, with experimental treatments focused on cold stress and shading. Such annotations are also called “conditions” represented by Cluster 2.

Weighted gene co-expression network analysis (WGCNA) was used to obtain global modules and cluster-specific modules from global samples and cluster samples. 23 co-expression modules were obtained based on the global expression profile, indicating the presence of complex and diverse co-expression relationships among genes (Table S5). Different numbers of co-expression modules were obtained based on the cluster expression profiles. Specifically, 12, 23, 22, and 14 co-expression modules were obtained based on Cluster 1 to Cluster 4 expression profiles, respectively (Table 1). The varying numbers of cluster-specific co-expression modules reflect changes in gene expression patterns under different cultivars, tissues, and experimental treatments.

Two perspectives of analysis were performed to elucidate the extent of differences in co-expression modules obtained from global samples and cluster samples: similarity analysis of module genes and internal consistency analysis of module expression profiles.

To investigate the similarity of co-expression modules obtained from global samples and cluster samples in WGCNA, the Fowlkes-Mallows score (FMS) was used as a metric. FMS is commonly used to compare the similarity of clusters or co-expression modules obtained from different samples or conditions. The FMS score ranges from 0 to 1, where a value closer to 1 indicates a higher similarity between the two data sets. Conversely, when FMS approaches 0, it indicates a low consistency between the two datasets. We observed that the FMS between the global module and cluster-specific modules ranges from 0.55 to 0.85 (Fig. 2C). Specifically, the similarity between the global module and the cluster-specific modules of Cluster 2 is low (Fig. 2C). Additionally, the cluster-specific module of Cluster 2 shows low similarity with the majority of other clusters’ specific modules (Fig. 2C). This suggests a higher level of uniqueness for Cluster 2, suggesting that the condition of Cluster 2 might employ a transcriptional program different from the other conditions.

We used the gene-module consistency coefficient (GMC) to assess the similarity between the expression profiles of genes and the average expression profile within a module. The GMC is essentially the correlation coefficient between the expression profiles of genes and the eigengene within a module. It ranges from−1 to 1, where a value greater than 0 indicates a positive correlation and a value less than 0 indicates a negative correlation. As expected, genes from the same module tend to have a GMC score larger than 0, as genes in the same module should be correlated (Fig. 2D). We observed that the median GMC of genes in the cluster-specific modules tends to be slightly higher than the GMC of genes in the global module, especially Cluster 2, which is significantly higher than the global module (Fig. 2D). This confirms that after classifying samples using k-means clustering, conducting co-expression analysis with cluster-specific samples generally improves accuracy, particularly for cluster samples that exhibit significant differences from the global samples, such as in Cluster 2.

### Understanding the condition-specificity of co-expression modules from two perspectives

In traditional WGCNA, after obtaining co-expression modules, there is often a biological functional annotation of the modules. However, here, we not only annotate the modules with functional information, but also calculate the conditional specificity of each module to each function. In this study, correlation difference value (CDV) is proposed as a measure of gene condition specificity. CDV is calculated as the difference between the gene-module consistency coefficient (GMC) of a gene in the cluster-specific module and its GMC in the global module. CDV values range from−2 to 2. A CDV value closer to 2 indicates a higher level of gene condition specificity, while a value closer to 0 indicates a higher level of conservation, as the average expression of the gene is more similar to the expression profile of the global module.

**Fig. 3:**
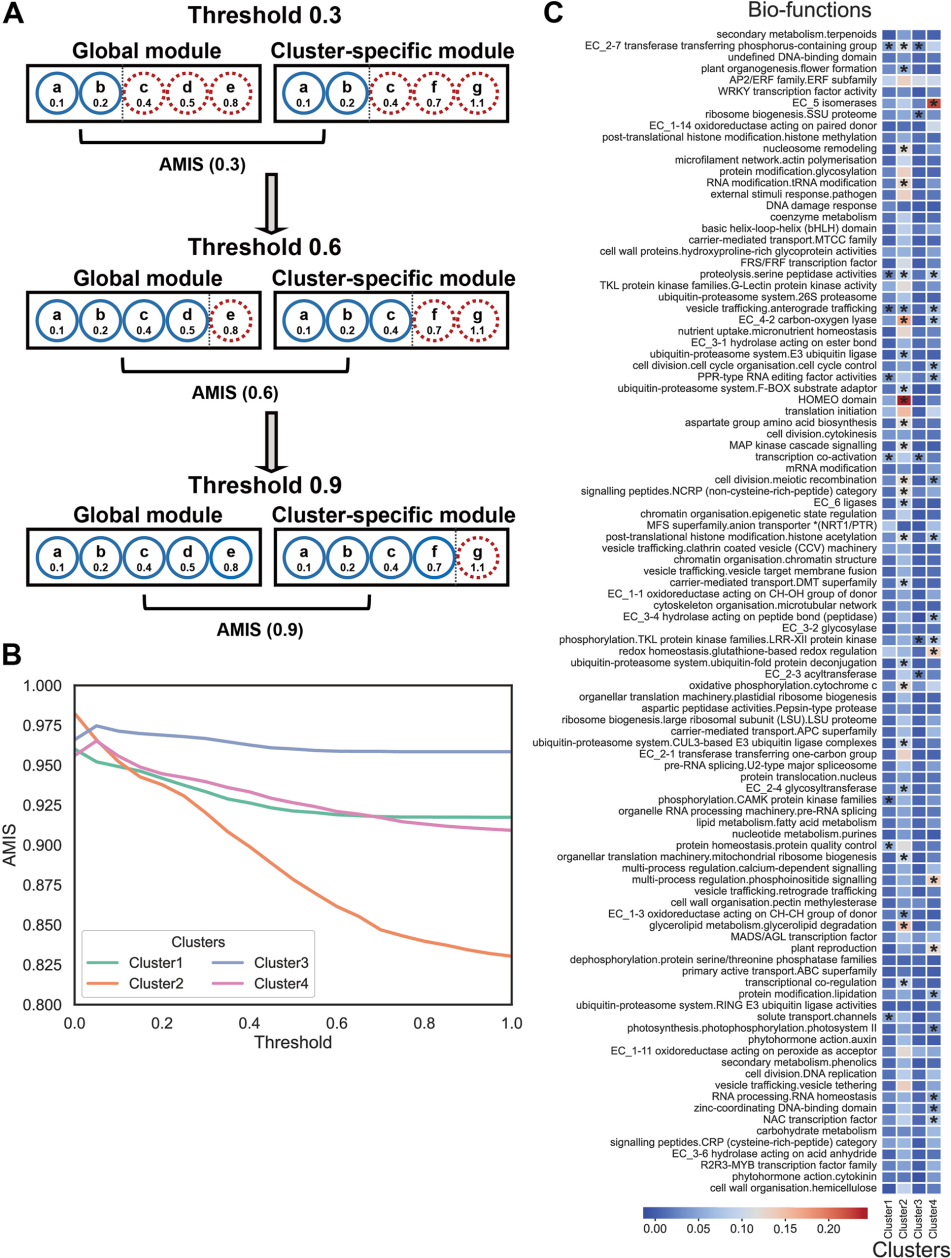
Relationship between correlation difference value (CDV) and condition specificity, and average CDV of different biological functions in different clusters. **A** Illustrative graph demonstrating the change in module similarity as the threshold increases from 0.3 to 0.9. **B** The impact of genes with different CDV on the similarity of global modules and cluster-specific modules. **C** CDV heatmap for each bio-function in each cluster. Cells marked with asterisks (*) indicate significant enrichment, and the color of the cells represents the average CDV

To explain the biological function of condition-specific genes, we analyzed a series of CDV thresholds ranging from 0 to 1. For each threshold, genes with CDV values higher than the threshold were considered cluster-specific, while genes with CDV values lower than the threshold were considered conserved. For each threshold, we calculated the similarity between the global module and the cluster-specific module after removing genes with values higher than the threshold (Fig. 3A). Therefore, lines in the line plot can be understood as follows: when the threshold is close to 0, there is a high similarity between the global module and the cluster-specific module. However, as the threshold increases from 0 to 1, genes with higher CDV values are included in both the global module and the cluster-specific module, resulting in a decrease in the similarity between them (Fig. 3B). In other words, genes with higher CDV values lead to lower similarity between the global module and the cluster-specific module, indicating higher condition specificity, while genes with lower CDV values have minimal impact on the similarity between the global module and the cluster-specific module. In Cluster 2, the similarity decreases most rapidly with increasing threshold, indicating that genes with high CDV values in Cluster 2 are more condition-specific (Fig. 3B).

Subsequently, the average CDV of genes with different biological functions in the four clusters revealed that Cluster 2, which was found to be least similar to the global module, has a higher proportion of genes with high average CDV values associated with specific biological functions (Fig. 3C). This further underscores the relationship between CDV and condition specificity. We observed that in Cluster 2, genes with CDV values higher than 0.2 are mainly enriched in biological functions such as “transcriptional co-regulation”, “MAP kinase cascade signalling” and several “ubiquitin-proteasome system”-related terms (Fig. 3C).

**Fig. 4:**
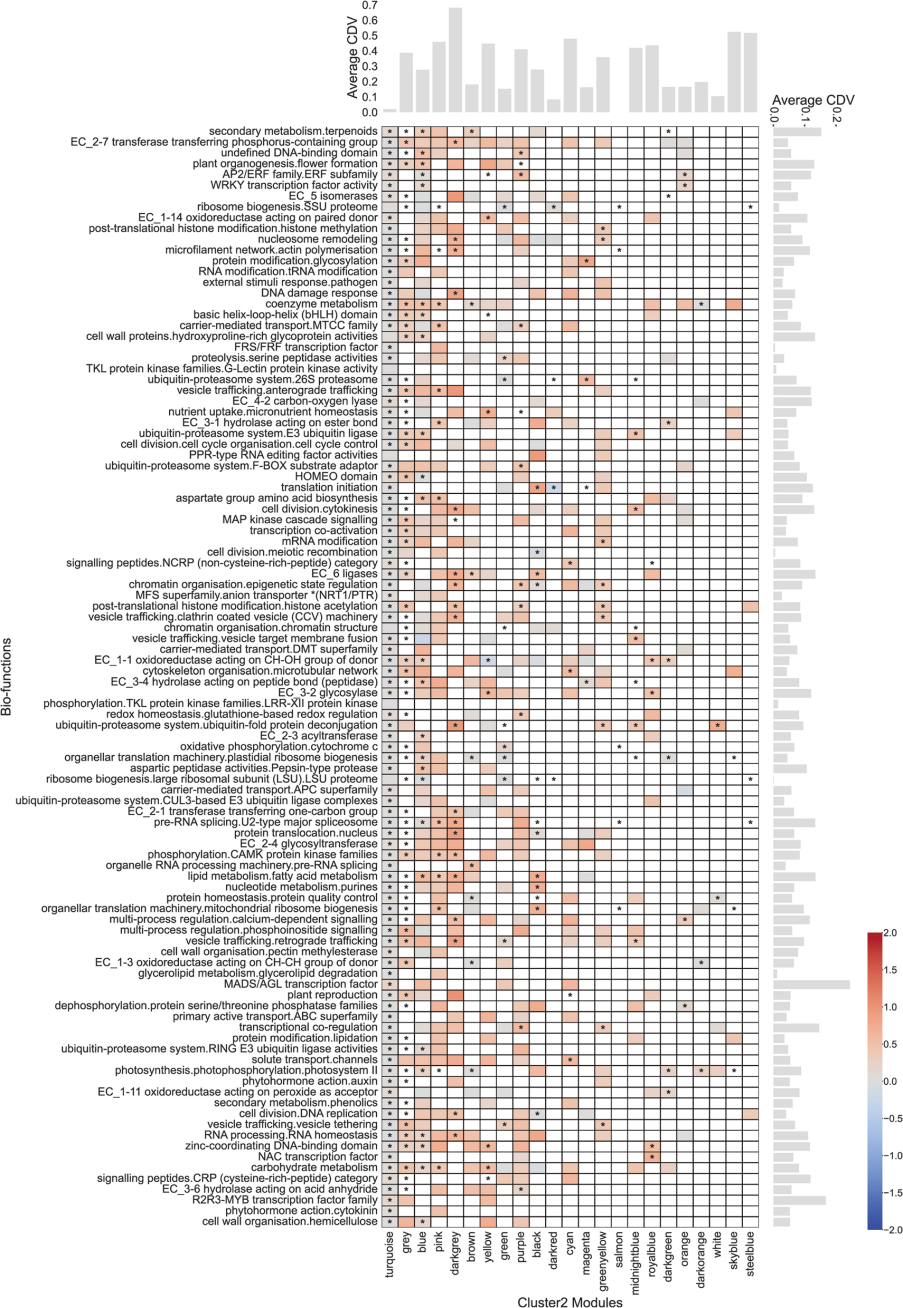
Correlation difference value (CDV) and functional enrichment heatmap corresponding to various biological functions for each co-expression module in Cluster 2. Cells marked with asterisks (*) indicate significant enrichment, and the color of the cells represents the average CDV. The blank cells in the figure indicate that the co-expressed module does not contain genes in that biological term or only genes with no CDV values

As discovered in the previous section, the Cluster 2 specific module is the least similar to the global module and is most likely to uncover condition-specific modules and biological functions. We analyzed Cluster 2 from two perspectives: gene condition specificity and biological function enrichment. By combining the average CDV (correlation difference value) heatmap, with the specific modules of Cluster 1 as the x-axis, and biological functions as the y-axis, and the results of significant biological function enrichment, we observed that the biological function with the highest average CDV are “MADS/AGL transcription factor”, “R2R3-MYB transcription factor family”, “secondary metabolism.terpenoids”, and “transcriptional co-regulation”, the module with the highest average CDV are “darkgrey”, “skyblue”, and “steelblue” (Fig. 4). Genes with high CDV values in the “purple” module are significantly enriched in biological functions such as “AP2/ERF family.ERF subfamily”, “transcriptional co-regulation”, “ubiquitin-proteasome system.F-BOX substrate adaptor”, and “redox homeostasis.glutathione-based redox regulation”, which has piqued our interest, prompting further investigation into this module (Fig. 4).

### Combining condition specificity and gene regulatory network reveals a series of transcription factors important in sustained cold stress

**Fig. 5:**
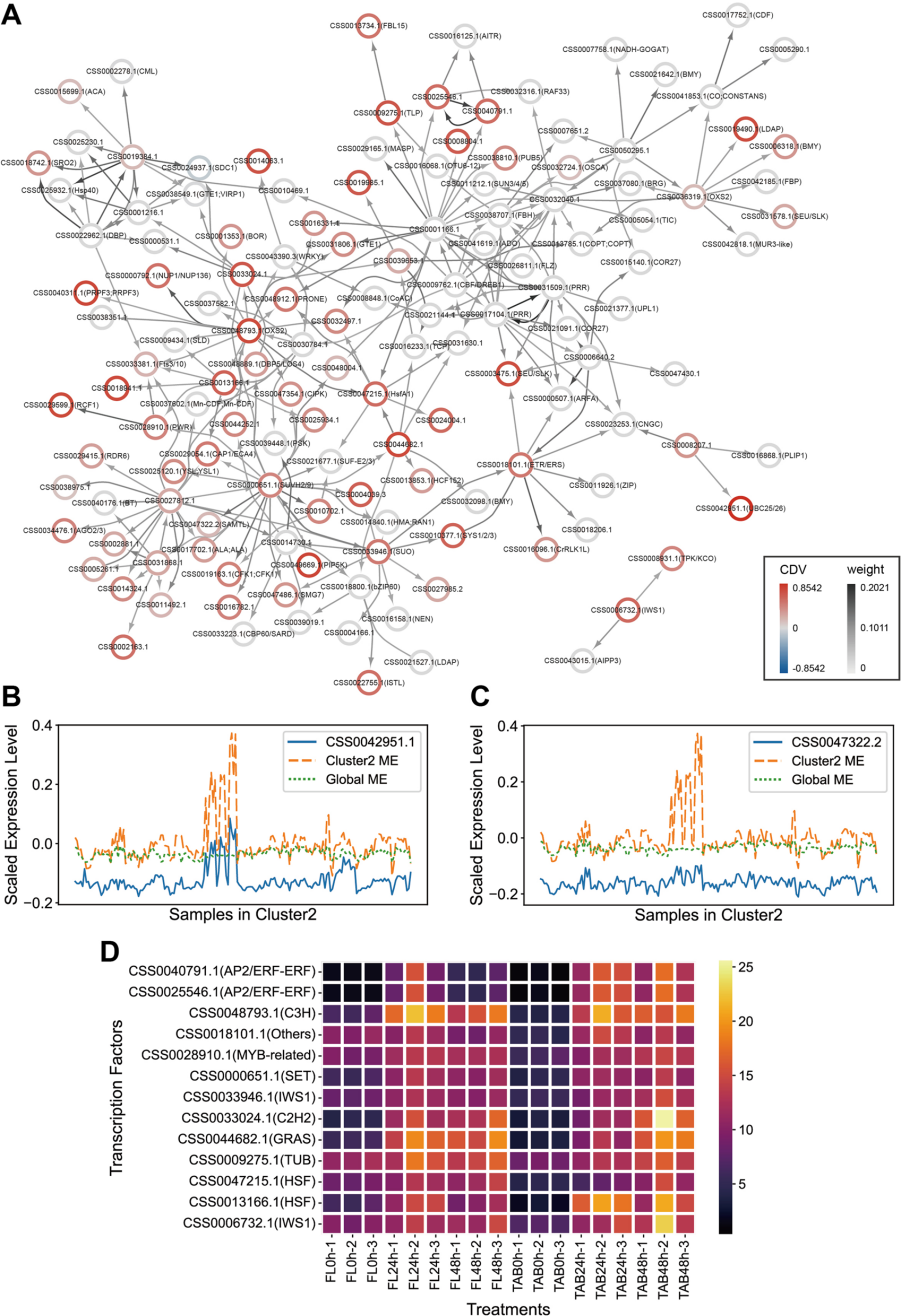
Gene regulatory network and comparison analysis of expression profiles. **A** Gene regulatory network of genes in the “purple” module of Cluster 2. The color intensity of the edges represents the weight between two nodes, and the color variation of the node borders represents the level of correlation difference value (CDV). **B** Comparison of the expression profile of gene CSS0042951.1 with the expression profiles of the eigengenes of the Cluster 2 module and the Global module. **C** Comparison of the expression profile of gene CSS0047322.2 with the expression profiles of the eigengenes of the Cluster 2 module and the Global module. **D** Expression levels of the high CDV transcription factor-encoding genes in the “purple” module of Cluster 2 under sustained low-temperature treatment in the first leaf (FL) and two leaves and a bud (TAB)

To predict the regulatory relationships between genes in the “purple” module and identify condition-specific co-expressed genes that could potentially explain the specificity of Cluster 2 under certain conditions, we constructed a gene regulatory network for the “purple” module and annotated the genes with their CDV. We observed that in the gene regulatory network of the “purple” module, there are 12 transcription factor encoding genes with CDV values greater than 0.4 (Fig. 5A). They encode transcription factors including AP2/ERF-ERF, C3H, SET, IWS1, C2H2, GRAS, TUB, HSF, and MYB-related (Fig. 5A). Additionally, there are 14 target genes regulated by transcription factors with CDV values greater than 0.6 (Fig. 5A). They encode proteins including PIP5K, PRPF3, RCF1, SEU/SLK, and glutathione S-transferase (Fig. 5A).

To further compare the differences between cluster-specific co-expression modules and global co-expression modules, and to demonstrate the role of CDV values, we selected a high CDV gene and a low CDV gene from the gene regulatory network of the “purple” module and plotted their expression profiles. By comparing the expression profile of the eigengene in the module where the gene in Cluster 2 is located (yellow line) with the expression profiles of the eigengene in the module where the gene in global is located (green line), we found that the eigengene of Cluster 2 module effectively capture the expression differences among samples within Cluster 2, while the eigengene of the global module do not represent the expression characteristics of the samples well (Fig. 5BC). This indicates that k-means clustering significantly enhances the accuracy of cluster sample co-expression analysis. Furthermore, the expression profile of gene *CSS0042951.1* with a higher CDV (0.8542) is more similar to the eigengene of Cluster 2 module, while the expression profile of gene *CSS0047322.2* with a lower CDV (0.1518) is difficult to distinguish and is more similar to the eigengene of the global module rather than the eigengene of Cluster 2 module, which intuitively demonstrates that genes with higher CDV values are more valuable for research (Fig. 5BC).

The eigengene expression profile of Cluster 2 in the “purple” module exhibits several very distinct peaks, corresponding to samples primarily concentrated in a sustained cold stress treatment experiment. We plotted the expression profiles of 12 transcription factor encoding genes with CDV values greater than 0.4 in this experiment. We observed that with increasing duration of cold treatment (from 0 h to 48 h), the expression levels of the majority of high CDV transcription factor encoding genes significantly increased, regardless of whether it was in the tea tree first leaf (FL) or two leaves and a bud (TAB) samples (Fig. 5D). Only two AP2/ERF-ERF encoding genes showed a significant increase in expression levels in two leaves and a bud (TAB) samples (Fig. 5D).

## Discussion

Tea plant (*Camellia sinensis*), being one of the world’s most important beverage crops, is known for its numerous secondary metabolites that contribute to the tea quality and health benefits. In order to characterize the biological functions of genes in tea plants, previous research has utilized a large-scale SRA data downloaded from NCBI to construct a gene co-expression network database known as TeaCoN (http://teacon.wchoda.com) [9].

However, when conducting co-expression analysis, more samples does not necessarily mean better results [10]. Researchers analyzed a dataset of *Escherichia coli* microarray data and found that subsets of the dataset performed better in inferring transcriptional regulatory networks [41]. The poor performance of the global network was attributed to increased multidimensional noise [11]. However, this issue can be mitigated by determining the optimal number of effective samples, for example, through a downsampling method that automatically groups samples using k-means clustering [12, 13].

In this study, we observed that the metadata entries (experimental treatments, tissues, and cultivars) of the SRA samples downloaded from NCBI were imbalanced (Fig. 1). This phenomenon has also been observed in other large-scale co-expression analysis studies [9]. The imbalanced sampling of global samples makes it difficult to represent specific research questions that require specific experimental conditions [42]. Therefore, in this study, a k-means clustering method was employed to automatically classify and organize all samples based on their gene expression patterns, forming distinct clusters (Figure S1; Fig. 2AB). By annotating these clusters, each cluster can represent specific conditions (Table 1).

Based on the comparative analysis from two perspectives, we observed significant differences between the global module and the cluster-specific modules in terms of gene composition (Fig. 2C). Furthermore, compared to the co-expression modules obtained from the global samples, the co-expression modules corresponding to the clustered samples indeed showed a significant improvement in accuracy (Fig. 2D). Specifically, under specific conditions, there was a higher similarity between the gene expression profiles and the average expression profiles of the modules they belonged to (Fig. 2D). Additionally, we found that the Cluster 2 specific module has the most unique gene composition and the most concentrated biological functions compared to the global module, which warrants further investigation (Fig. 2CD).

Although the co-expression analysis of the clustered samples has higher accuracy, it does not mean that the analysis of the global samples becomes meaningless. On the contrary, by combining the co-expression analysis of the clustered samples with that of the global samples, we can obtain more valuable information. In this study, a correlation difference value (CDV) was proposed to explain the condition specificity of a gene by comparing the correlation between the gene expression profile and the average expression profile of the cluster-specific module, and the correlation between the gene expression profile and the average expression profile of the global module under specific conditions. CDV has been demonstrated in this paper to reflect the impact of a gene on the similarity between the cluster-specific module and the global module (Fig. 3AB). Genes with higher CDV values exhibit higher condition specificity and are worth further investigation.

In this study, through the investigation of the specific condition Cluster 2, we identified a co-expression module “purple” highly associated with cold stress. In its gene regulatory network, a series of genes encoding high-CDV transcription factors were significantly upregulated in the continuously cold-treated tea plant leaves and buds (Fig. 5A). These transcription factor-encoding genes include AP2/ERF-ERF, C3H, SET, IWS1, C2H2, GRAS, TUB, HSF, and MYB-related factors, most of which have been extensively linked to cold stress response in tea plants in numerous studies [43–46].

Researchers have found in past studies that GST and HSF interact to some extent in cellular antioxidant stress responses and coping with external pressures [47]. In this study, we observed that a glutathione S-transferase (GST) encoding gene, *CSS0018941.1*, with a high CDV (0.7153), is regulated by a heat shock factor (HSF) encoding gene, *CSS0013166.1*, with a high CDV (0.5926), implying the involvement of GST and HSF interaction in the antioxidant defense system of tea plants under sustained cold stress, aiding in the clearance of harmful compounds and oxidative stress products within cells. Heatmaps of the expression profiles of CSS0013166.1, a homologous gene *AT1G67970.1* in *Arabidopsis* (E-value = 1e−26), and *CSS0018941.1*, a homologous gene *AT1G10370.1* in *Arabidopsis* (E-value = 4e−04), were plotted in ePlant (https://bar.utoronto.ca/eplant/.) [48]. We found that both *AT1G67970.1* and *AT1G10370.1* exhibited an upregulation trend under various abiotic persistent stresses, including sustained cold treatment, further confirming the findings in this study (Figure S3).

### Supplementary Information

**Supplementary Material 1.**

**Supplementary Material 2.**

**Supplementary Material 3.**

**Supplementary Material 4.**

**Supplementary Material 5.**

**Supplementary Material 6.**

**Supplementary Material 7.**

**Supplementary Material 8.**

**Supplementary Material 9.**

## Data Availability

The raw RNA sequencing data used in this study are available from the NCBI Sequence Read Archive (SRA) under the accession numbers listed in Table S1. No sequencing data were generated during this study.

## Abbreviations

WGCNA: weighted gene co-expression network analysis
CDV: Correlationdifference value
CDS: Coding sequences
TPM: Transcripts per million
FDR: False discovery rate
FMS: Fowlkes-Mallows score
AMIS: Adjusted mutualinformation score
GMC: Gene-module consistency coefficient
PCC: PEARSONcorrelation coefficient
t-SNE: t-Distributed Stochastic Neighbor Embedding
