# Supplementary figures and images for: A method for mining condition-specific co-expressed genes in Camellia sinensis based on k-means clustering
Source: BMC Plant Biol. 2024 May 8;24:373. doi: 10.1186/s12870-024-05086-5 (PMC11077725; doi:10.1186/s12870-024-05086-5)

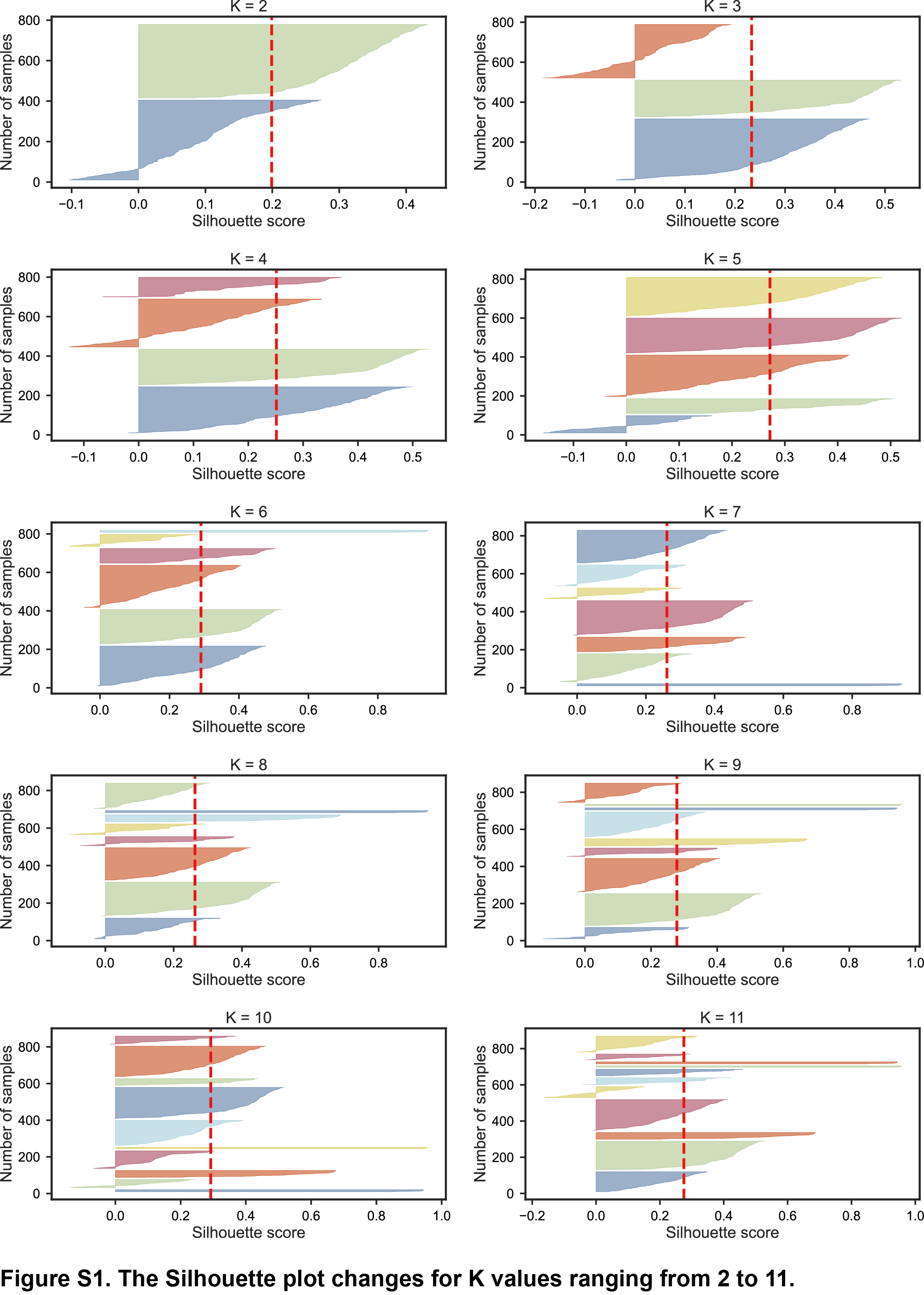

Supplement: Supplementary file 4 — Supplementary Material 4. [file 12870_2024_5086_MOESM4_ESM.jpg]

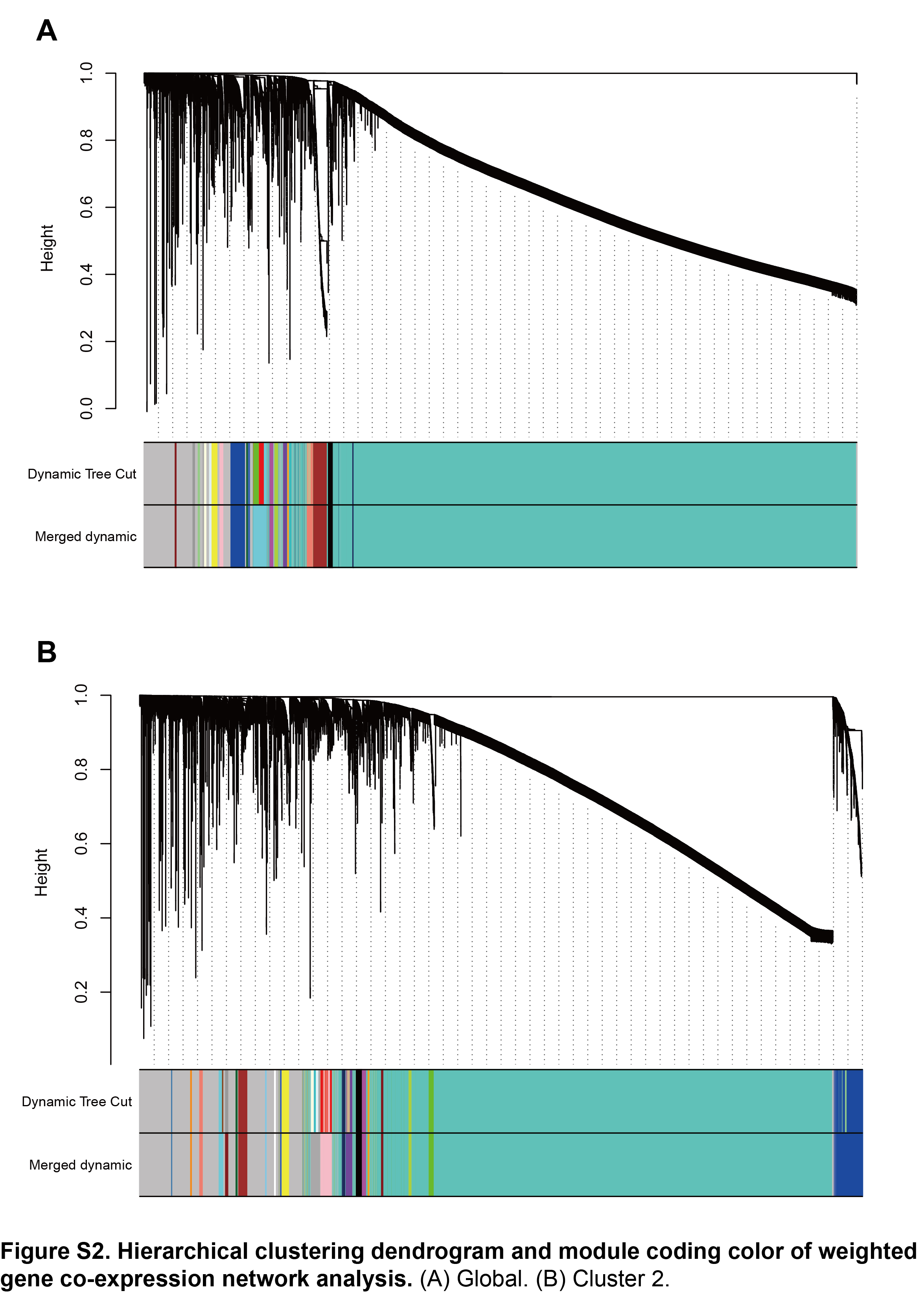

Supplement: Supplementary file 5 — Supplementary Material 5. [file 12870_2024_5086_MOESM5_ESM.jpg]

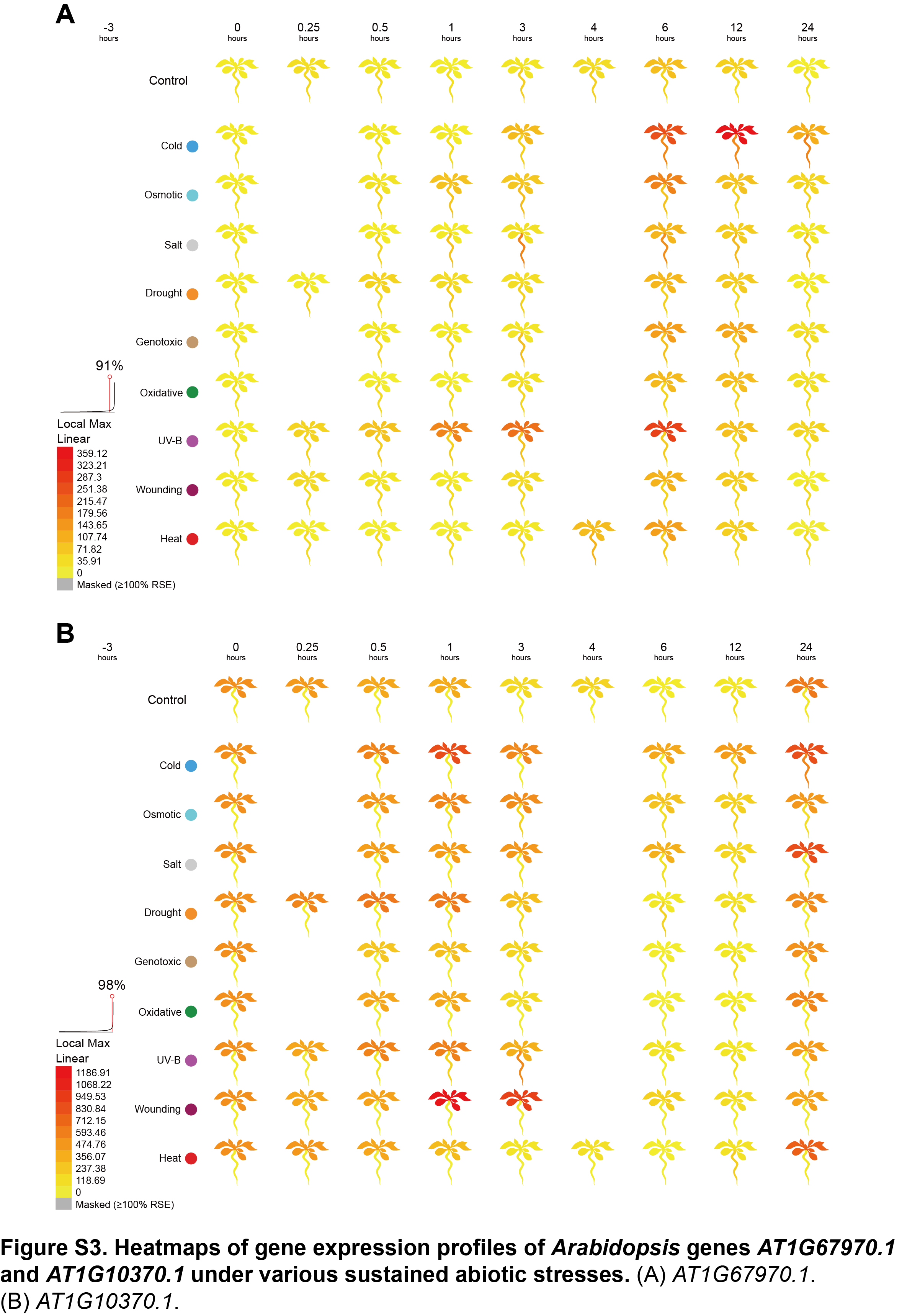

Supplement: Supplementary file 9 — Supplementary Material 9. [file 12870_2024_5086_MOESM9_ESM.jpg]
